# Microbiology of acute bacterial dacryocystitis: a tertiary institutional experience in South Australia

**DOI:** 10.1007/s10792-024-03236-0

**Published:** 2024-06-26

**Authors:** Akash Gowda, Jessica Y. Tong, Dinesh Selva

**Affiliations:** 1 South Australian Institute of Ophthalmology, The University of Adelaide, Adelaide, SA 5005 Australia; 2 Department of Ophthalmology, Royal Adelaide Hospital, Adelaide, SA 5000 Australia; 3 Discipline of Ophthalmology and Visual Sciences, The University of Adelaide, Adelaide, SA 5000 Australia

**Keywords:** Acute dacryocystitis, *Staphylococcus aureus*, Antibiotic, Sensitivity

## Abstract

**Purpose:**

To provide a comprehensive microbiological profile of bacterial dacryocystitis in South Australia. By identifying the specific microorganism and antibiotic susceptibility, this study intends to aid ophthalmologists in choosing appropriate empirical antibiotic therapies and development of evidence-based clinical guidelines.

**Method:**

A retrospective study was conducted at the Royal Adelaide Hospital (RAH) over five years (2018–2023) of patients with acute dacryocystitis. The study included 43 patients, and data encompassed demographic information, clinical presentation, microbiological analysis, management, and outcomes. Patients with chronic dacryocystitis were excluded.

**Results:**

Among the 43 patients included in the study (female 28 (65%), mean age: 64 years old), the most common clinical features were pain (74%) and swelling (70%). Organisms were identified in 49% of patients, with the predominant bacteria being *Staphylococcus aureus* (42%), *Streptococcus* species (19%), and *Escherichia coli* (8%). *Aggregatibacter* species (8%), *Morganella morganii* (4%), *Enterobacter cloaceae* (4%), *Hafnia alvei* (4%), mixed anaerobes (4%), *E coliforms* (4%) and *Pseudomonas aeruginosa* (4%) were also identified. The most frequently prescribed empirical antibiotics were amoxicillin-clavulanic acid (50%), flucloxacillin (33%) and cefalexin (18%).

**Conclusion:**

The microbiological trends of acute dacryocystitis have largely remained consistent, with a predominance of Gram positive organisms. This is the most recent profile analysis of acute dacryocystitis in South Australia and will help form evidence-based clinical guidelines.

**Supplementary Information:**

The online version contains supplementary material available at 10.1007/s10792-024-03236-0.

## Introduction

Acute dacryocystitis is the most common disease of the lacrimal drainage system [1, 2]. Bacterial aetiologies comprise between 6 and 95% of all dacryocystitis presentations [1]. If not treated appropriately and promptly, there is risk of progression of the infection resulting in orbital cellulitis, abscess, meningitis and cavernous sinus thrombosis [1]. The major risk factors for developing acute dacryocystitis include: female gender, nasolacrimal duct obstruction, nasal septal deviation and dacryolith [3]. Whilst there are several studies of the microbiological profile of dacryocystitis [2–9], there are no recent studies looking into the microbiological profile of acute dacryocystitis in Australia, with the last one being conducted in 2005 [8]. The most common pathogens isolated in dacryocystitis, both acute and chronic, are *Staphylococcus aureus*, *Streptococcus pneumoniae*, *Haemophilus influenzae*, *Serratia marcescens* and *Pseudomonas aeruginosa*, however, the proportions in which these are found differ depending on geographical region [3]. This study aims to highlight the microbiological landscape of dacryocystitis, with the primary objective of characterising the spectrum of bacteria associated with acute dacryocystitis in South Australia. Overall, this study provides updated findings that may guide appropriate empirical antibiotic choice and inform clinical guidelines.

## Method

This was a single-centre retrospective study of patients diagnosed with acute dacryocystitis at the Royal Adelaide Hospital over a 5-year period from 2018 to 2023. Patients with incomplete clinical records, no microbiological analysis conducted or those lost to follow-up were excluded. Data was sourced from paper and electronic medical records. Data collected included demographics (age at presentation, sex, past medical history and previous antibiotic use), clinical presentation, radiology findings, microbiological analysis, course of management and clinical outcomes. Microbiological specimens were obtained at first presentation to the ophthalmology clinic. They were obtained by either direct lacrimal aspirate or by wiping a swab across the lower conjunctival cul-de-sac near the medial canthus and punctum by applying pressure over the lacrimal sac area. Purulent material was then transported in Amies medium if conjunctival swab was taken or sterile pot if a lacrimal aspirate was obtained, for culture and sensitivity testing.

Samples were plated and incubated on Columbia horse blood agar (HBA) (5% CO_2_ at 35 ± 2 °C for 36 h), Columbia CNA horse blood agar (CAN) (O_2_ at 35 ± 2 °C for 36 h), chocolate + Neisseria medium (Choc/NIMM agar) (5% CO_2_ at 35 ± 2 °C for 36 h) and anaerobic plain agar plate (anaerobically at 35 ± 2 °C for 84 h) [Edwards group, Narellan, NSW, Australia]. Where significant growth occurred, organisms were identified using matrix-assisted laser desorption/ionization time of flight (MALDI-TOF) mass spectrometry (MALDI Biotyper Sirius IVD system, Bruker Daltonic GMBH, Bremen, Germany). Antimicrobial susceptibility testing (AST) was performed using standard disc diffusion on Mueller Hinton E (MHE) or Mueller Hinton EUCAST agar with horse blood and NAD (MHND) [Edwards group, Narellan, NSW, Australia]. Where applicable, minimum inhibitory concentrations (MICs) were determined by automated microdilution broth testing (BD-Phoenix), Becton Dickinson, Sparks, Maryland, USA). Where applicable, the interpretation of antibiotic susceptibility results was performed according to European Committee on Antimicrobial susceptibility testing (EUCAST) guidelines.

All research was conducted in accordance with the Declaration of Helsinki and was approved by Central Adelaide Local Health Network Human Research Ethics Committee.

## Results

This study included 43 patients (Female: 28, Male: 15) between May 2018 and May 2023 (inclusive) from one tertiary oculoplastic unit. The mean age was 63 years (range: 22–90 years). Relevant comorbidities included: diabetes mellitus (9/43, 21%), pre-existing dacryolith (1/43, 2%), nasolacrimal duct stenosis and/or obstruction (17/43, 40%), nasal septal deviation (5/43, 12%), previous trauma to orbit and/or nose (1/43, 2%), neoplasm (2/43, 5%), previous surgery to orbit and/or nose (3/43, 7%). All cases were unilateral. The most common clinical features were: pain (33/43, 77%), swelling (31/43, 72%) and epiphora (24/43, 56%). Other notable clinical features were: discharge (18/43, 42%), localised erythema (12/43, 28%), subjective visual changes (7/43, 16%) and fever (5/43, 12%). 40/43 (93%) patients had a first episode of acute dacryocystitis whilst 3/43 (7%) patients had an episode of acute on chronic dacryocystitis.

Of the 31 cases where swabs or cultures were taken, 4/31 (13%) of cases had a lacrimal aspirate specimen sent, whilst the rest (27/31, 87%) had conjunctival swabs of discharge. Of those who had cultures taken, on microbiological analysis, 21/31 (68%) patients had organisms identified from a lacrimal aspirate or swab, including polymicrobial cultures. No organism was cultured in 10/31 (32%) patients. There was no record of a swab or aspirate being taken for 12/43 (28%) cases. Of the 21 cases with positive cultures, a total of 26 bacterial pathogens were grown. Gram positive organisms comprised 16/26 (62%) of isolates; 9/26 (35%) were Gram negative organisms. Organisms included: *Staphylococcus aureus* (11/26, 42%), *Streptococcus pneumoniae* (3/26, 21%), *Escherichia coli* (2/26, 8%), *Aggregatibacter* species (2/26, 8%), *Streptococcus pyogenes* (1/26, 4%), *Streptococcus anginosus* (1/26, 4%), *Morganella morganii* (1/26, 4%), *Enterobacter cloaceae* (1/26, 4%), *Hafnia alvei* (1/26, 4%), mixed anaerobes (1/26, 4%), *E coliforms* (1/26, 4%) and *Pseudomonas aeruginosa* (1/26, 4%). Of the *Staphylococcus aureus* identified on culture, 2/11 (22%) were methicillin-resistant *Staphylococcus aureus* (MRSA). A single pathogen was identified in 16/21 (76%) patients, whilst 5/21 (24%) had polymicrobial cultures. Of the five polymicrobial cultures, there was a combination of *Streptococcus pneumoniae* (1/10, 10%), *Streptococcus pyogenes* (1/10, 10%)*, Streptococcus anginosus* (1/10, 10%), mixed anaerobes (1/10, 10%), *Morganella morganii* (1/10, 10%), *Aggregatibacter* species (1/10, 10%), *Hafnia alvei* (1/10, 10%) and *Staphylococcus aureus* (1/10, 10%).

Empirical antibiotics were given in 40/43 (93%) of cases, of which 20/40 (50%) were admitted for a period of intravenous (IV) antibiotics. These regimens included flucloxacillin (12/20, 60%) amoxicillin/clavulanic acid (7/20, 35%), ceftriaxone (5/20, 25%), cefazolin (3/20, 15%), piperacillin/tazobactam (1/20, 5%), metronidazole (1/20, 5%) and vancomycin (1/20, 5%). The remaining 20/40 (50%) who were treated as outpatients were given a course of amoxicillin/clavulanic acid (13/20, 65%), cefalexin (4/20, 20%), azithromycin (1/20, 5%), flucloxacillin (1/20, 5%) and clindamycin (1/20, 5%). The 3/43 (7%) for whom empirical antibiotics were not prescribed, 2/3 (67%) were previously given antibiotics which were not documented in the electronic medical record, 1/3 (33%) was previously on doxycycline long-term and this antibiotic was continued.

Tables 1 and 2 demonstrate the sensitivities of commonly tested antimicrobials in the Gram positive (Table 1) and Gram negative (Table 2) organisms identified in this study. Gram positive organisms were all sensitive to vancomycin, TMP/SXT and chloramphenicol. 93% of Gram positive organisms on which Clindamycin was tested (*Staphylococcus aureus*, *Streptococcus pneumoniae*, *Streptococcus pyogenes*) were sensitive. 83% Gram positive organisms on which cefalexin were tested (*Staphyloccocus aureus*, *Streptococcus pyogenes*) were sensitive. 82% of *Staphylococcus aureus* organisms cultured were sensitive to flucloxacillin. 100% of *Streptococcus pneumoniae* were sensitive to amoxicillin-clavulanic acid. While all *S, pneumoniae, S. pyogenes* and *S. pyogenes* were susceptible to penicillin and amoxicillin, overall only 36% of Gram positive organisms causing dacryocystitis were susceptible to penicillin or amoxicillin.

**Table 1:** Summary of sensitivity rate of commonly tested antimicrobials of Gram positive organisms––*Staphylococcus aureus* and *Streptococcus* species

		Sensitivity (%) against selected antibiotic								
		Ampicillin	Penicillin	Flucloxacillin	Cefalexin	Chloram	Amox-Clav	Clinda	TMP/SXT	Vancomycin
Organism	n									
Staphylococcus aureus*	11	0%	10%	82%	82%	100%	N/A	91%	100%	100%
MRSA	2	0%	0%	0%	0%	100%	N/A	50%	100%	100%
*Streptococcus pneumoniae*	3	100%	100%	N/A	N/A	100%	100%	100%	100%	100%
*Streptococcus pyogenes*	1	N/A	100%	N/A	100%	N/A	N/A	100%	N/A	100%^
*Streptococcus anginosus*	1	N/A	100%	N/A	N/A	N/A	N/A	N/A	N/A	100%^

n, Number of Organisms; N/A, Not Applicable (Not tested on culture); Chloram, Chloramphenicol; Amox-Clav, Amoxicillin-Clavulanic Acid; Clinda, Clindamycin; TMP/SXT, Trimethoprim/Sulfamethoxazole; MRSA, Methicillin-resistant *Staphylococcus aureus*

^*^Includes 2 cases of Methicillin-Resistant *Staphylococcus aureus*

^Not tested but vancomycin resistance has not been detected

**Table 2:** Summary of sensitivity rate of commonly tested antimicrobials of Gram negative organisms

		Sensitivity (%) against selected antibiotic							
		Ampicillin	Cefalexin	Ceftriaxone	Amox-Clav	Gentamicin	Ciprofloxacin	TMP/SXT	Piperacillin-Tazobactam
Organism	n								
*Escherichia coli*	2	100%	N/A	100%	100%	100%	100%	N/A	N/A
*Aggregatibacter* species	2	100%	N/A	100%	N/A	N/A	N/A	N/A	N/A
E coliforms*	1	N/A	N/A	N/A	100%	N/A	N/A	N/A	N/A
*Morganella morganii*	1	0%	0%	N/A	0%	100%	100%	100%	100%
*Enterobacter cloacae*	1	0%	0%	0%	0%	100%	100%	100%	100%
*Hafnia alvei*	1	0%	0%	100%	0%	100%	100%	100%	100%
*Pseudomonas aeruginosa*	1	N/A	N/A	N/A	N/A	100%	100%	N/A	100%

n, Number of Organisms; N/A, Not Applicable (Not tested on culture); Chloram, Chloramphenicol; Amox-Clav, Amoxicillin-Clavulanic Acid; TMP/SXT, Trimethoprim/Sulfamethoxazole

^*^Microbiology report did not clarify type of *E coliforms*, only testing Amoxicillin-Clavulanic Acid

All Gram negative organisms identified were susceptible to gentamicin, TMP/SXT, piperacillin/tazobactam and ciprofloxacin. 50% of Gram negative organisms on which ceftriaxone was tested (*Escherichia coli, Aggregatibacter species, Enterobacter cloacae, Hafnia alvei*) were susceptible. Only *Escherichia coli* and *E coliforms* were universally susceptible to amoxicillin-clavulanic acid whilst *Morganella morgagnii*, *Enterobacter cloacae* and *Hafnia alvei* were all resistant.

The majority of patients [31/43, 72%] had complete resolution of dacryocystitis with no further episodes. Recurrence occurred in 8/43 [19%], and 4/43 [9%] were lost to follow up. Of those who experienced recurrence, 4 grew bacteria on their cultures, of which 2/4 [50%] were *Staphylococcus aureus*, 1/4 [25%] was *Streptococcus pneumoniae* and 1/4 [25%] was *Pseudomonas aeruginosa*. One patient had persisting symptoms with oral flucloxacillin therapy but was found to have MRSA and transitioned antibiotics to clindamycin which eventually resulted in resolution of dacryocystitis.

## Discussion

Acute dacryocystitis likely occurs due to bacterial overgrowth in the stagnant fluid of the lacrimal sac [4]. It can become a sight- or life-threatening infection with the potential to progress to orbital cellulitis, meningitis, and/or cavernous sinus thrombosis [10]. There are limited studies evaluating the microbiological profile of acute dacryocystitis, with the last study analysing dacryocystitis, both acute and chronic, in Australia being published in 2005 [8]. By providing an update on the microbiological landscape in dacryocystitis, targeted treatment can benefit the patient, particularly at a time of growing concerns regarding antimicrobial resistance [11].

The spectrum of bacterial pathogens and antibiotic susceptibility are reasonably consistent across various geographical regions[2]. Microbiological studies of dacryocystitis in Australia [8], North America [5], Iran [6, 7], China [1] and India [2, 4, 9] report that *Staphylococcus aureus* is the most common pathogen implicated in acute dacryocystitis, followed by *Streptococcus* species (including *Streptococcus pneumoniae, Streptococcus pyogenes* and *Streptococcus anginosus*). Razavi et al.’s earlier study from Iran identified *Staphylococcus epidermidis* as the most common cause of acute dacryocystitis [12], while *Staphylococcus aureus* is the predominant in more contemporary studies. *S. epidermidis* and other coagulase negative staphylococci are considered part of normal skin flora and therefore not routinely reported by our institution. Previous studies from Australia [8], China [1], India [4] and Iran [6] showed *Pseudomonas aeruginosa* and *Haemophilus influenzae* being common Gram negative isolates in dacryocystitis. In contrast, in this study, *Escherichia coli* and *Aggregatibacter* were the most common Gram negative organisms, with no cultures identifying *Haemophilus influenzae*. In this study, Gram positive isolates tended to predominate in acute dacryocystitis, which is consistent with much of the literature [2–9]. One of the unique cases in this series was a patient who had an acute dacryocystitis secondary to *Hafnia alvei*, a Gram negative enteric and oropharyngeal bacillus [13]. Infections caused by *Hafnia alvei* can occasionally be responsible for serious nosocomial and community acquired infections [14]. Cahill & Burns was the only paper in the literature that described finding more Gram negative isolates than Gram positive in patients with acute dacryocystitis, but this study was published in 1993 and the microbiology of dacryocystitis has likely changed since then [15]. Briscoe et al. found a predominance of Gram negative organisms, but the authors focused on purulent acute and chronic dacryocystitis, which likely represented a population with more virulent organisms [16].

MRSA infections are of increasing concern to clinical practitioners [17]. Risk factors include recent hospitalization, nursing home residence, use of injectable drugs, antibiotic usage, and chronic illness [18] should be screened appropriately. In this retrospective study, two patients had MRSA, representing 18% of *Staphylococcus aureus* infections causing dacryocystitis, which is similar to the incidence of MRSA for all ophthalmic infections (22%) reported from the same institution in 2012 [19]. MRSA has been found to be significantly associated with acute dacryocystitis as opposed to chronic presentations [5]. Dacryocystitis secondary to MRSA can be challenging to manage as it is more likely to be refractory to conservative treatment, and a definitive dacryocystorhinostomy (DCR) is often required for resolution [17]. In this study where there were two MRSA cases of acute dacryocystitis, one patient achieved resolution following an oral antibiotic course of clindamycin, while the other required an admission for IV vancomycin, a DCR within 1 week of admission, and was discharged onto oral trimethoprim-sulfamethoxazole. Both patients had resolution of dacryocystitis with no further recurrences documented.

In this study, the most common initial oral antibiotics prescribed were amoxicillin/clavulanic acid (13/20, 65%) and cefalexin (4/20, 20%). The current guidelines in Australia recommend cefalexin as the empirical treatment for dacryocystitis [20]. For the pathogens isolated in this study, 77% would have been susceptible to cefalexin (tested on 15 pathogens) and 82% would have been susceptible to flucloxacillin (tested on 11 pathogens). On the 17 pathogens in which TMP/SXT was tested, 100% were sensitive. Given the predominance of Gram positive organisms in dacryocystitis, antibiotics such as cephalosporins and amoxicillin/clavulanic acid may appear to be appropriate choices for initial antibiotic therapy, however, 34% of patients had their antibiotic treatment changed based on culture results. Furthermore, flucloxacillin alone may have too narrow a therapeutic range for empiric therapy as Gram negative organisms are not uncommonly identified in this condition. Flucloxacillin in combination with ceftriaxone is recommended for inpatients for coverage of periorbital cellulitis in the context of dacryocystitis, as is supported by local therapeutic guidelines [21]. This regimen would cover most of the organisms identified to cause dacryocystitis in this study, with the exception of the two cases of MRSA infection. Regarding Gram negative isolates in patients with purulent dacryocystitis, quinolones (e.g. ciprofloxacin), cephalosporins and aminoglycosides (e.g. gentamicin) have shown good coverage [4, 6]. If clinicians are concerned about a resistant organism, they may consider using amoxicillin/clavulanic acid as next-line empiric therapy [21]. TMP/SXT should also be considered if there is concern regarding MRSA or Gram negative organisms resistant to amoxicillin-clavulanic acid due to pathogens (e.g. *Morganella morganii*, *Hafnia alvei*, *Klebsiella aerogenes*, *Proteus vulgaris*) that constitutively produce amp C beta-lactamases [22].

The results obtained from the study was compared with the typical antibiotic resistance profile in antibiograms provided by our institution for common ophthalmic, otolaryngologic and upper respiratory pathogens identified in the same time period (Supplement 1). With the exception of TMP/SXT, the resistance profile of the organisms causing dacryocystitis in our study were consistent with those of the larger dataset representing typical antimicrobial susceptibilities in South Australia for similar samples.

There were several limitations of this study including its small sample size and retrospective nature. An additional limitation is the use of conjunctival swabs for specimen collection rather than lacrimal aspiration. Whilst there is a theoretical increase in risk of contamination when utilising conjunctival swabs [3], all attempts were made in this study to collect discharge from the region of the medial canthus and punctum as a representation of discharge secondary to a refluxable mucocele. Furthermore, performing a lacrimal aspirate can be a very painful procedure for the patient in the setting of an acute dacryocystitis. Nevertheless, it provides a snapshot of one tertiary Oculoplastic unit’s experience with acute dacryocystitis and the prevalence of microorganisms over a 5-year period. Another caveat in this study is that there was a notable proportion of cultures not being available for analysis, either due to culture-negative samples or no sample being taken (22/43, 51%). This was due to a number of factors, including commencement of empirical treatment in the community prior to presenting at our institution thus resulting in sterile aspirates, or no purulent discharge available to be swabbed.

## Conclusion

In conclusion, we present the microbiological profile of bacterial dacryocystitis in a major tertiary centre in Australia. This study has demonstrated that microbiological profiles remain relatively consistent across geographical regions. The sensitivities of the bacteria implicated in dacryocystitis can guide empirical antibiotic therapy in the future. Currently, cefalexin is considered first-line empiric therapy for acute dacryocystitis, with amoxicillin-clavulanic acid or TMP/SXT considered if there is initial failure of therapy or high risk of resistance or MRSA. MRSA continues to be a growing concern and such patients are at increased risk of treatment failure resulting in prolonged illness. This study can be used in conjunction with other future epidemiological and microbiological analyse to inform guidelines and choice of empirical antibiotics.

## Supplementary Information

Below is the link to the electronic supplementary material.

Supplementary file1 (XLSX 26 kb)

## Data Availability

No datasets were generated or analysed during the current study.
